# Experimental Characterization of the Isomer-Selective Generation of the Astrochemically Relevant Hydroxymethylene Radical Cation (HCOH^•+^/DCOH^•+^)

**DOI:** 10.1021/acs.jpclett.4c02374

**Published:** 2024-10-24

**Authors:** Vincent Richardson, Luke Alcock, Nicolas Solem, David Sundelin, Claire Romanzin, Roland Thissen, Wolf D. Geppert, Christian Alcaraz, Miroslav Polášek, Brianna R. Heazlewood, Quentin Autret, Daniela Ascenzi

**Affiliations:** † Department of Physics, The Oliver Lodge, University of Liverpool, Oxford St, Liverpool L69 7ZE, United Kingdom; ‡ Université Paris-Saclay, CNRS, Institut de Chimie Physique, UMR8000, 91405 Orsay, France/Synchrotron SOLEIL, L’Orme de Merisiers, 91190 Saint Aubin, France; § Department of Physics, Stockholm University, Roslagstullsbacken 21, S-10691 Stockholm, Sweden; ∥ J. Heyrovský Institute of Physical Chemistry of the Czech Academy of Sciences, Dolejšškova 2155/3, 182 23 Prague, Czechia; ⊥ Department of Physics, University of Trento, Via Sommarive 14, 38123 Trento, Italy

## Abstract

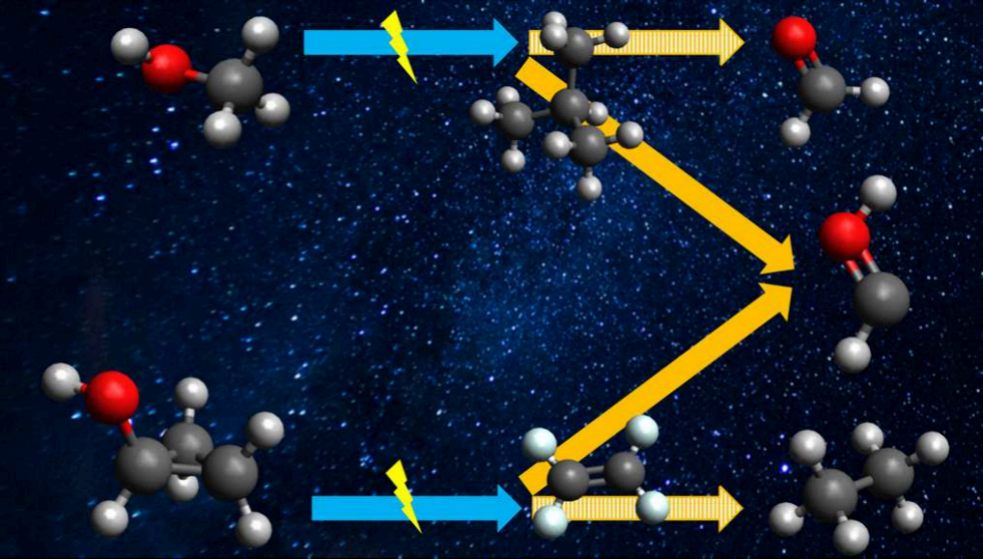

Interest in the observation and characterization of organic isomers in astronomical environments has grown rapidly with an increase in the sensitivity of detection techniques. Accurate modeling and interpretation of these environments require experimental isomer-specific reactivity and spectroscopic measurements. Given the abundance of formaldehyde (H_2_CO) in various astrophysical objects, the properties and reactivities of its cation isomers H_2_CO^•+^ and HCOH^•+^ are of significant interest. However, for the hydroxymethylene radical cation HCOH^•+^ (and its isotopologue DCOH^•+^), detailed reactivity studies have been limited by the lack of suitable experimental methods to generate this isomer with high purity. Here, potential approaches to the isomer-selective generation of HCOH^•+^ and DCOH^•+^ are characterized through differential reactivity measurements. While the dissociative photoionization of cyclopropanol (c-CH_2_CH_2_CHOH) is determined to be unsuitable, the dissociation of methanol-*d*_3_ (CD_3_OH) allows for the formation of DCOH^•+^ with a fractional abundance of >99% at photon energies below 14.8 eV. These results will allow future spectroscopic and reactivity measurements of HCOH^•+^/DCOH^•+^ to be conducted, laying the groundwork for future detection and incorporation into models of the interstellar medium.

Following the determination of the relative abundances of the HCO^+^/HOC^+^ and HCN^•+^/HNC^•+^ isomeric ion pairs in the interstellar medium (ISM),^1−4^ there has been a growing acknowledgment of the importance of isomerism and experimental isomer-specific reactivity measurements in astrochemistry.^5,6^ Isomerization barriers that are sufficiently small to allow for isomers to exist in equilibrium under terrestrial conditions can be prohibitive at the lower temperatures present in many astrochemical environments, such as dark clouds. Conversely, for some species, the higher energy isomer is found to be the most abundant, a situation that is unlikely under terrestrial conditions. As such, the differing chemical and spectroscopic properties of isomers necessitate isomer-specific laboratory measurements in order to accurately model and interpret the chemistry of astronomical environments.

Isomer-selective reactivity has been shown to be of particular importance to the modeling of planetary and satellite atmospheres, with a notable example being the contribution of different [C_3_H_3_]^+^ isomers to the chemistry of Titan, the largest moon of Saturn.^7^ Furthermore, isomer-selective generation is also important for fundamental gas-phase kinetics measurements,^8−11^ as the differing reactivities of isomers can provide important insights into the interrelationship between structure and reactivity.^12,13^ This is especially significant for small radical cations where subtle changes in structure can lead to significantly different reactivity.^14−19^

Neutral formaldehyde (H_2_CO) has long been known to be present in the ISM,^20^ where it is routinely detected in large and varied range of astrophysical objects, from diffuse clouds^21^ to protoplanetary disks^22^ and protoplanetary nebulae^23^ (see Appendix A in ref (24) for a list of objects where formaldehyde and its isotopologues have been detected). With an ionization energy (IE) of 10.9 eV,^25,26^ it is anticipated that H_2_CO will be readily ionized by UV photons, cosmic rays, and high-energy electrons. As the anticipated product ions, H_2_CO^•+^ and HCOH^•+^, are yet to be explicitly detected in the ISM, likely due to the highly transient nature of radical cations even in such low density environments, the need for precise laboratory-based studies of these species is clear. While the formaldehyde radical cation (H_2_CO^•+^) is the most stable [CH_2_O]^•+^ isomer, the *trans* and *cis* HCOH^•+^ isomers are only 0.3 and 0.44 eV higher in energy, respectively.^27^ A third isomer, COH_2_^•+^, will probably play a minor role in cold environments since it is more than 2 eV higher in energy than the H_2_CO^•+^ and HCOH^•+^ isomers,^28^ and so it is not considered further here.

Importantly, the barrier to isomerization between H_2_CO^•+^ and *trans* HCOH^•+^ of 1.97 eV is higher in energy than that for the fragmentation into HCO^+^ plus H, meaning that interconversion over this barrier is highly unlikely. Furthermore, a recent computational study^29^ determined the tunneling lifetime of the HCOH^•+^ isomer to be on the order of thousands of years at representative ISM temperatures. Given this, formation mechanisms such as the protonation of HCO,^30^ radical hydrogen abstraction from H_2_COH^+^,^31^ or dissociative charge transfer reactions with methanol^32,33^ have the potential to form a mix of the two [CH_2_O]^•+^ isomers under the low temperature conditions typical of the ISM.

While the production of H_2_CO^•+^ (e.g., by ionization of H_2_CO) has been amply investigated,^34,35^ the generation of HCOH^+•^ (and its isotopologue DCOH^+•^) is less straightforward and has been the subject of significant research interest due to its implications for both astrochemistry and fundamental reaction dynamics. Furthermore, it is notable that the characterization of the isomer-selective generation of neutral HCOH has been reported only very recently.^36^ While the dissociative ionizations of cyclopropanol (c-CH_2_CH_2_CH(OH)),^37,38^ methanol (CH_3_OH),^38−40^ and trideuterated methanol (CD_3_OH)^17,38^ have been suggested as suitable methods for generating the HCOH^+•^ isomer, or its partially deuterated isotopologue DCOH^•+^, no quantitative experimental characterization of the isomeric purity has yet been performed. Here, we present experimental results on the characterization of the isomeric purity of HCOH^+•^ and DCOH^+•^ formed from both cyclopropanol and CD_3_OH for use in future ion-molecule reactivity studies.

Measurements have been performed using a guided ion beam tandem mass spectrometer, allowing for ions formed in the source chamber to be mass-selected prior to introduction into the reaction cell. By monitoring the pressure in the cell and the intensity of different parent and product mass channels, absolute cross sections (CSs) and branching ratios (BRs) can be obtained for the reactions of different parent ions. Measurements are recorded as a function of both the energy of the photons used in the dissociative photoionization process and the collision energy available to the reactants. Tunable VUV sources, in this case the DESIRS beamline^41^ of the SOLEIL synchrotron radiation facility, allow for a high level of control over the photon energy. This, in turn, allows for the tuning of the internal energy of the ions formed and, by extension, the fragmentation dynamics. Full experimental details, including a discussion of the uncertainties associated with the measurements reported here, are given in the Supporting Information.

For a source generating a mixture of two different species A and B (either isomers or entirely different chemical species), the observed reaction CS of the mixture, σ_T_, can be described by the following equation
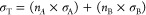
1where *n*_A_ and *n*_B_ are the fractions of A and B, respectively, while σ_A_ and σ_B_ are the absolute reaction CSs of pure samples of A and B, respectively. If σ_A_ and σ_B_ are known, then *n*_A_ and *n*_B_ can be determined for a given σ_*T*_. Here, we have employed this approach to determine the isomeric purity, i.e., the relative yields of the HCOH^•+^ and H_2_CO^•+^ isomers from different generation methods. However, due to the lack of isomer-selective reactivity data for HCOH^•+^ and DCOH^•+^, this quantification must be performed through the subtraction of the H_2_CO^•+^ fractional abundance, for which isomer-selective generation is well-established.

The ionization of cyclopropanol has previously been shown to produce an intense *m*/*z* 30 fragment at 1–2.5 eV above the ionization threshold of 9.10 eV.^37,42^ Though the *m*/*z* 30 fragment was initially assigned exclusively to the formation of HCOH^•+^ in combination with C_2_H_4_,^37^ a more recent study^42^ provided an alternative assigment—attributing it instead to the formation of C_2_H_6_^•+^ in combination with CO, though we note that the HCOH^•+^ isomer is not discussed in this study. The reattribution to C_2_H_6_^•+^ plus CO was based on the threshold energy (1.47 eV above the 9.10 eV ionization energy of cyclopropanol) being in agreement with the calculated dissociation limit of 10.57 eV, while fragmentation into H_2_CO^•+^ plus C_2_H_4_ has a higher dissociation limit of 11.35 eV. A further study^38^ noted that, while the *m*/*z* 30 fragment is a doublet of C_2_H_6_^•+^ and [CH_2_O]^•+^, the [CH_2_O]^•+^ component is consistent with the HCOH^•+^ isomer.

In order to benchmark our results against this previous study,^42^ the appearance energies (AEs) of the relevant fragments have been measured from their respective photoionization efficiency (PIE) curves, with the results for both the parent ion (*m*/*z* 58) and the *m*/*z* 29 and 30 fragments shown in Figure 1. For the *m*/*z* 29 and 30 fragments this has been performed via linear threshold extrapolation of the PIE curve,^43−46^ while, due to the lack of a well-defined onset, the AE for the *m*/*z* 58 parent ion has been determined as the first point above noise. We note that the AE values given here, and elsewhere in this work, are used only for comparison with AEs determined previously in the literature and not to derive relevant thermochemical values. For a complete discussion on the fitting of AE thresholds, please refer to Roithová et al.,^43^ Ruscic,^47^ and the references therein. For the *m*/*z* 58 ion, the measured AE of 9.15 ± 0.12 eV is in good agreement with the previously measured IE of 9.10 eV.^42^ Similarly, the AE of the *m*/*z* 30 fragment of 10.58 ± 0.04 eV is in excellent agreement with the previous measurement of 10.57 eV.^42^ Finally, the AE for the *m*/*z* 29 channel, which has previously been assigned to a mix of C_2_H_5_^+^ and HCO^+^, is measured as 10.94 ± 0.05 eV, in reasonable agreement with the previously reported AEs of 10.75 and 10.88 eV for the two channels, respectively.^42^ Notably, the *m*/*z* 29 channel shows a sharp rise in intensity above ∼12 eV, in agreement with the observation of the previous study,^42^ where it was assigned in part to the opening of the channel leading to the formation of C_2_H_5_^+^ in combination with CO and H^•^, which has an AE of 11.49 eV.^42^

**Figure 1:**
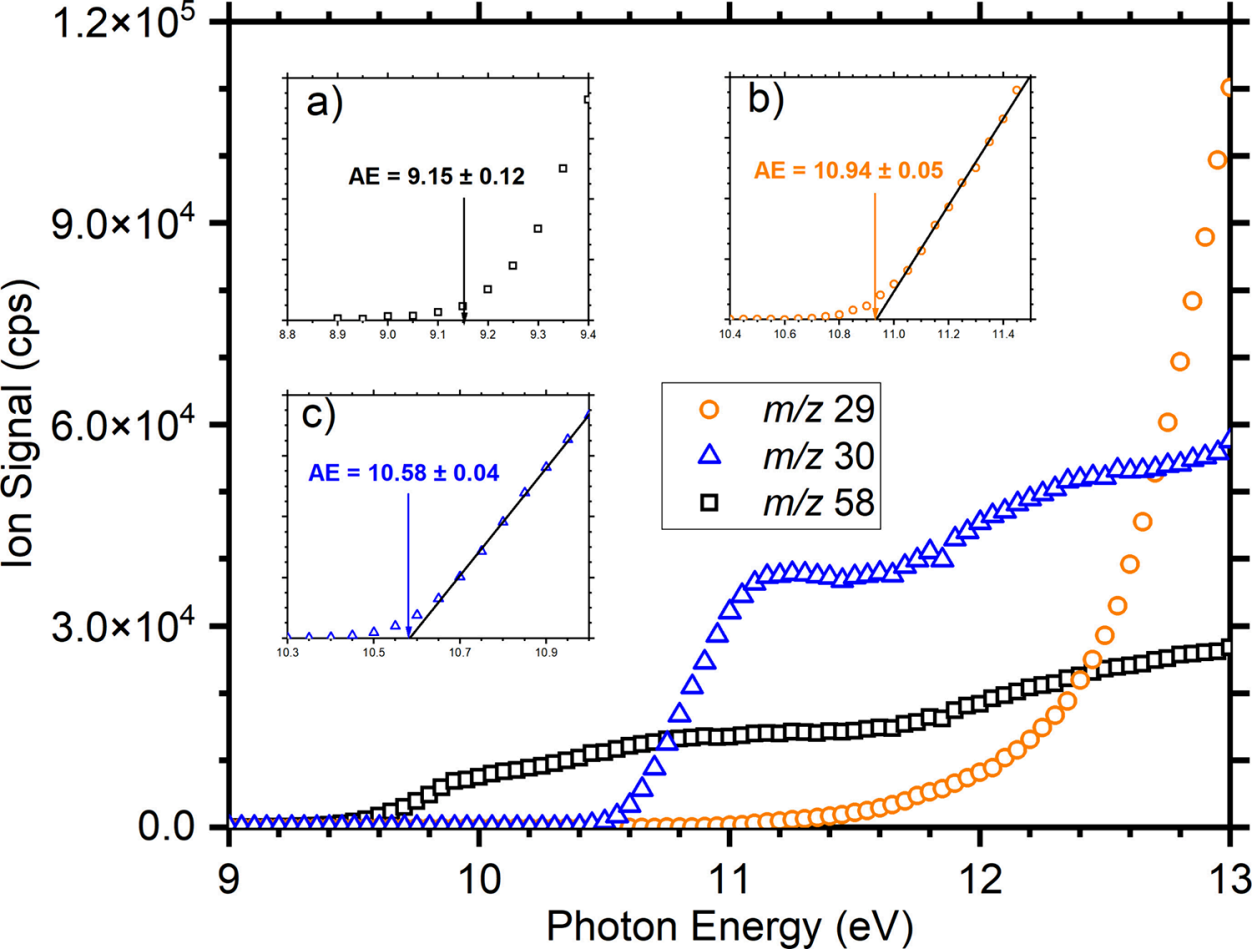
Photoionization efficiency (PIE) curves for the dissociative photoionization of cyclopropanol giving [C_3_H_6_O]^+^ (*m*/*z* 58, black squares), [CHO]^+^/C_2_H_5_^+^ (*m*/*z* 29, orange circles), and [CH_2_O]^•+^/C_2_H_6_^•+^ (*m*/*z* 30, blue triangles). Insets a), b), and c) show the threshold regions for the corresponding *m*/*z* values, along with the assigned appearence energies (AEs).

In order to reduce any internal energy effects while maintaining a sufficient ion yield at *m*/*z* 30, the reactivity experiments discussed below are performed at a photon energy of 11.2 eV. C_2_D_4_ is chosen as the neutral reactant to disentangle the presence of C_2_H_6_^•+^ from that of [CH_2_O]^•+^. The reaction of C_2_H_6_^•+^ with C_2_H_4_ has been studied previously,^48^ and the sole *m*/*z* 28 product (C_2_H_4_^•+^), corresponding to the charge transfer, is observed with a rate of (1.15 ± 0.12) × 10^–9^ cm^3^·molecule^–1^·s^–1^. C_2_D_4_ is chosen in preference to its undeuterated equivalent (C_2_H_4_) to simplify the analysis by partially deuterating product species that would otherwise be equivalent as, while the charge transfer reaction is also energetically accessible for the H_2_CO^•+^ ion, a previous study of the reaction of this ion with C_2_D_4_ observed not only the charge transfer product (*m*/*z* 32, C_2_D_4_^•+^), but also a distinctive combination of proton (*m*/*z* 33, C_2_D_4_H^•+^) and hydrogen atom transfer (*m*/*z* 29, HCO^+^) channels.^16^ As the BRs, and their relative collision energy dependencies, have been reported in detail previously,^16^ consideration of the reaction of the *m*/*z* 30 fragment with C_2_D_4_ therefore allows for discrimination between the relative contributions of the C_2_H_6_^•+^, H_2_CO^•+^, and HCOH^•+^ ions. Details of the data treatment are provided in the Supporting Information, with the resulting absolute CSs as a function of the collision energy given in Figure 2.

**Figure 2:**
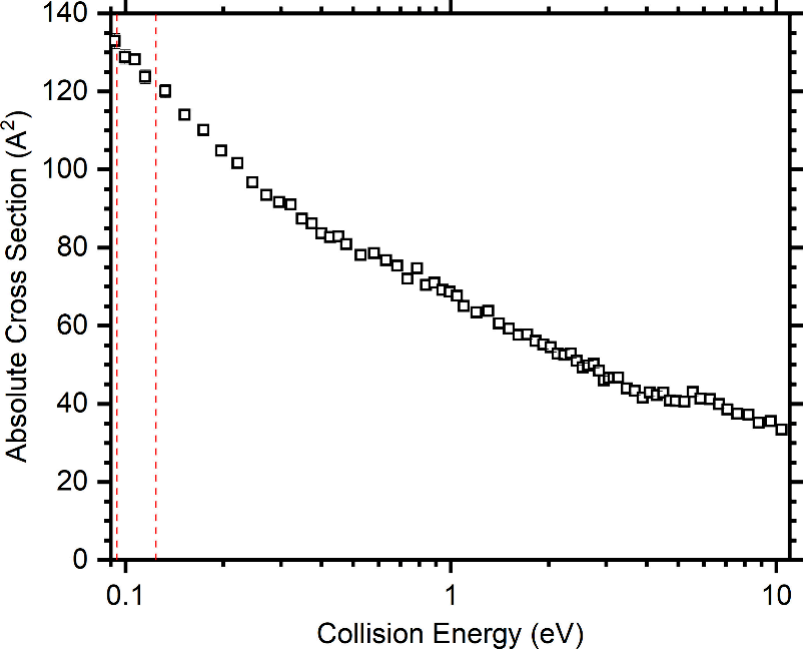
Summed absolute cross sections (CSs) as a function of the collision energy for the *m*/*z* 32 charge transfer product C_2_D_4_^•+^ and the corresponding secondary products at *m*/*z* 46, 62, 74, and 78, following the reaction of the *m*/*z* 30 fragment from the dissociative photoionization of cyclopropanol with C_2_D_4_ at *E*_phot_ = 11.2 eV. The red dashed lines represent the collision energy uncertainty range for the cross section used for the rate calculations, as detailed in the text.

Given CSs at a fixed collision energy, energy-dependent total rate constants can be obtained from the following expression
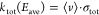
2where σ_tot_ is the absolute reaction CS and ⟨*v*⟩ is the average relative velocity obtained from the collision energy.^49,50^ In order to compare the results of our measurements with those of the C_2_H_6_^•+^ ion recorded using an ion cyclotron resonance (ICR) mass spectrometer,^48^*k*_tot_(*E*_ave_) is calculated from the experimental data at a collision energy of 0.11 ± 0.02 eV, with the obtained *k*_tot_(*E*_ave_) value of (1.48 ± 0.55) × 10^–9^ cm^3^·molecule^–1^·s^–1^ being in good agreement with the literature value of (1.15 ± 0.12) × 10^–9^ cm^3^·molecule^–1^·s^–1^ for the reaction of the C_2_H_6_^•+^ ion.

While we do observe minor products at *m*/*z* 33, 60, and 61 that are indicative of the presence of [CH_2_O]^•+^, we conclude that the majority of the *m*/*z* 30 fragment ion signal does indeed correspond to the formation of C_2_H_6_^•+^. This conclusion is reached through comparison of the observed *m*/*z* 32 (and subsequent secondary reaction) CSs with those of previous studies on the reaction of C_2_H_6_^•+^. In this way, we are able to determine that the lower limit, within error, for the fraction of C_2_H_6_^•+^ present is 0.57, with an upper limit for the fraction of HCOH^•+^ of 0.10. The dissociative ionization of cyclopropyl alcohol is therefore disregarded as a suitable method for the selective generation of the HCOH^•+^ ion.

The dissociative photoionization of trideuterated methanol (CD_3_OH) was first suggested by Berkowitz^40^ as being preferable to the dissociative ionization of fully hydrogenated methanol due to the ability to separate the D_2_CO^•+^ (*m*/*z* 32) and DCOH^•+^ (*m*/*z* 31) fragments by their mass-to-charge ratios.^17,38^ However, as there is the potential for H/D exchange prior to ejection, as evidenced by the experimental observation of the D_2_COD^+^ fragment (*m*/*z* 34) with an AE of 11.85 eV that is below that of 12.60 eV for the *m*/*z* 31 fragment,^51^ the potential for isobaric contamination from DHCO^•+^ has to be considered.

As with cyclopropanol, PIE curves for the *m*/*z* 30 ([CDO]^+^/[^13^CHO]^+^) and 31 ([CDHO]^•+^/[^13^CDO]^+^) fragments have been measured and are presented in Figure 3. The AE of the *m*/*z* 31 channel has been determined, again using linear threshold extrapolation, to be 12.52 ± 0.04 eV, in reasonable agreement with the value of 12.6 eV recorded previously.^51^ Due to a small contamination from H_2_CO in the source chamber from previous measurements, the *m*/*z* 30 fragment channel has a baseline of ∼100 cps, with the appearance energy of the DCO^+^ channel determined from the first point above noise as 12.90 ± 0.09 eV.

**Figure 3:**
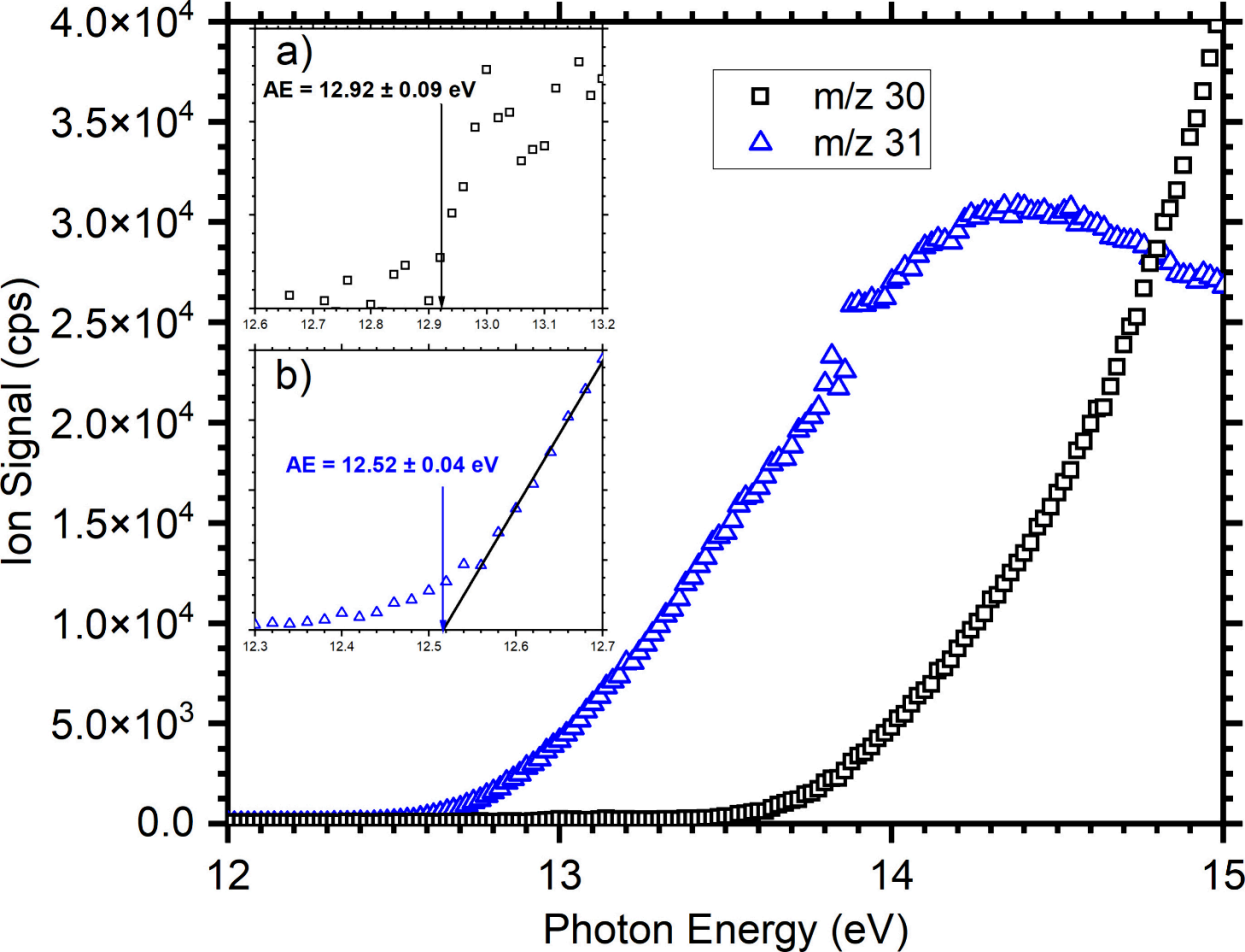
Photoionization efficiency (PIE) curves for the dissociative photoionization of CD_3_OH giving DCO^+^/COD^+^ (*m*/*z* 30, black data) and DCOH^•+^/DHCO^•+^ (*m*/*z* 31, blue data). Insets a) and b) show the threshold regions for the corresponding *m*/*z* 30 and 31 fragments, along with the assigned appearence energies (AEs).

For the characterization of any DHCO^•+^ impurity in the *m*/*z* 31 channel from deuterated methanol, isobutane (CH(CH_3_)_3_) has been selected as the reactant neutral because its IE of 10.68 ± 0.11 eV^26^ is between the IEs of DHCO (the IEs of H_2_CO and D_2_CO being 10.889 ± 0.003 eV^25,26^ and 10.908 ± 0.003 eV,^25^ respectively) and HCOH, which has an IE of 8.91 ± 0.02 eV.^28^ This means that the charge transfer process from DHCO^•+^ will be exothermic, while the charge transfer from DCOH^•+^ will be endothermic (and therefore closed at low collision energies in the absence of internal excitation). For this process, eq 1 can be rewritten as follows
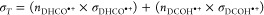
3where *n*_DHCO^•+^_ and *n*_DCOH^•+^_ are the isomeric fractions of DHCO^•+^ and DCOH^•+^, respectively, with σ_DHCO^•+^_ and σ_DCOH^•+^_ being the reaction CSs for the charge transfer reaction with CH(CH_3_)_3_ for DHCO^•+^ and DCOH^•+^, respectively. Given the endothermicity of the charge transfer process for the DCOH^•+^ isomer with isobutane, σ_DCOH^•+^_ can be assumed to be zero in this case, thereby allowing the fraction of DHCO^•+^ present to be determined as follows:
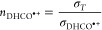
4In order to determine σ_DHCO^•+^_, and under the assumption that there are no kinetic isotope effects so that σ_DHCO^•+^_ is equal to σ_H_2_CO^•+^_, we have measured CSs for the reaction of H_2_CO^•+^ with isobutane as a function of both photon and collision energy. As mentioned above, this determination of *n*_DCOH^•+^_ through subtraction of *n*_DHCO^•+^_ is necessitated by the current lack of isomer-selective DCOH^•+^ reactivity data.

For the purpose of a reactivity study with CH(CH_3_)_3_, H_2_CO^•+^ ions have been generated via direct ionization of neutral formaldehyde (H_2_CO), in turn formed via pyrolysis of paraformaldehyde at 60 °C. The obtained PIE curve around the threshold region for the *m*/*z* 30 (H_2_CO^•+^) channel is shown in Figure S3 of the Supporting Information, from which an AE for the *m*/*z* 30 mass channel of 10.88 ± 0.04 eV has been obtained via linear threshold extrapolation of the PIE curve.^43−46^ This is in excellent agreement with the literature IE of H_2_CO of 10.889 ± 0.003 eV.^25,26^

Absolute reaction CSs as a function of the collision energy have been measured at a photon energy of 11.0 eV in order to limit the impact of internal energy effects while ensuring sufficient reactant ion flux to allow for accurate reactivity measurements. The primary reaction product is that at *m*/*z* 58 (CH(CH_3_)_3_^•+^), corresponding to charge transfer, with absolute CSs for this channel, σ_H_2_CO^•+^_, as a function of the collision energy shown in Figure 4. The step function visible in CS for both channels at a collision energy of ∼0.9 eV is due to the “L3 effect”, a key indicator of a charge transfer process, with further details given in the Supporting Information. This effect has not been corrected for here as the isomeric fraction is determined from the ratio of the two cross sections, which should remain constant. Although a range of other minor channels are also observed, these are not relevant to the characterization of the isomeric purity and so are not discussed further here.

**Figure 4:**
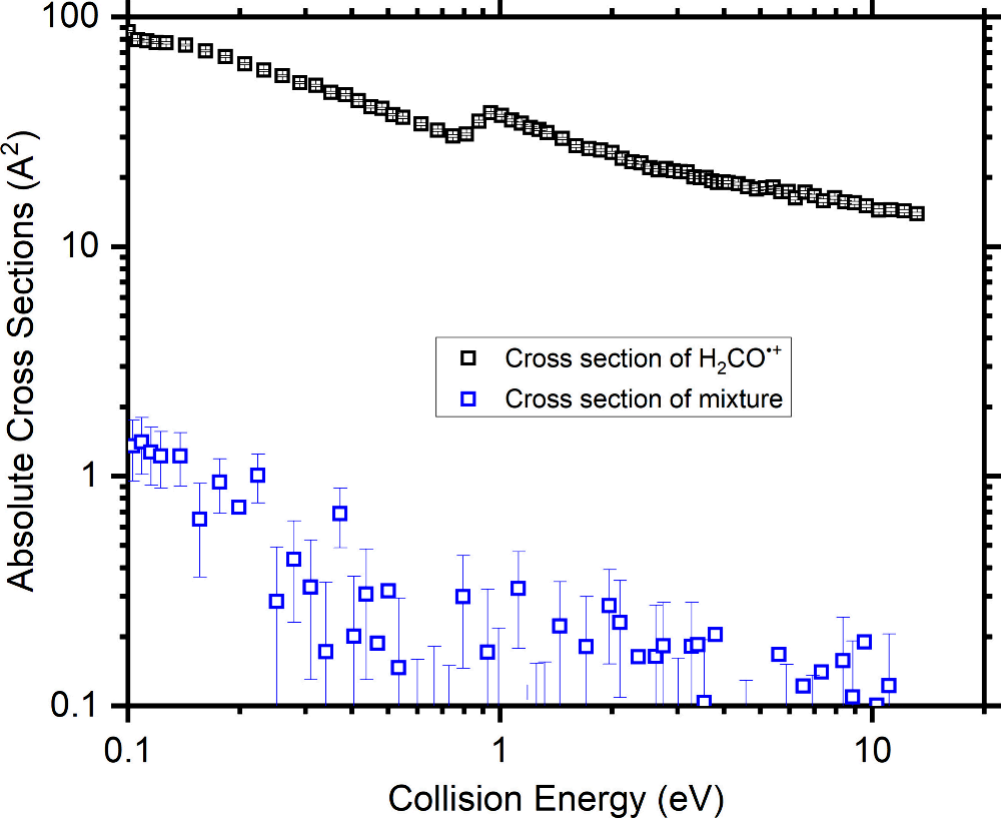
Absolute cross sections (CSs) as a function of the collision energy for the *m*/*z* 58 charge transfer product (CH(CH_3_)_3_^•+^) of the reaction with CH(CH_3_)_3_. Data for the reaction of H_2_CO^•+^ generated via direct photoionization of H_2_CO^•+^ (black squares) have been recorded at a photon energy of 11.0 eV, while those for the mixture of DCOH^•+^ and DHCO^•+^ ions generated from the dissociative ionization of CD_3_OH (blue squares) have been recorded at a photon energy of 13.07 eV.

In order to ensure that σ_DHCO^•+^_ is constant over the 11–12.5 eV photon energy, the CSs for the reaction product at *m*/*z* 58 have also been recorded as a function of the photon energy, with the results shown in Figure S4 of the Supporting Information. From this, we note that the measured absolute CSs are approximately independent of the photon energy with only a very slight decrease at higher photon energies, indicating that the absolute CS for this process can be treated as constant with regard to the internal energy of the reactant ions.

Having recorded σ_H_2_CO^•+^_, and therefore inferred σ_DHCO^•+^_, σ_*T*_ values have been obtained as a function of the collision energy at a photon energy of 13.07 eV, above the 12.52 eV AE of the *m*/*z* 31 fragment channel, with the results shown in Figure 4. As with the reaction of H_2_CO^•+^, we have also measured the CS for this channel as a function of the photon energy, with results shown in Figure S5 of the Supporting Information. No photon energy dependence for σ_*T*_ is observed, allowing the isomeric purity values obtained from the σ_*T*_ values recorded at a photon energy of 13.07 eV to be used throughout the 13–15 eV photon energy range.

The values of *n*_DHCO^•+^_ obtained from the measured σ_DHCO^•+^_ and σ_*T*_ values as a function of the collision energy are shown in Figure 5, while numerical values of *n*_DHCO^•+^_, σ_DHCO^•+^_, and σ_*T*_ at selected collision energies of 0.16, 2.0, and 11.0 eV are given in Table 1. From this, we are able to determine an upper limit for the DHCO^•+^ fraction at this photon energy of 2.3%, with the average *n*_DHCO^•+^_ value over this collision energy range being 0.7 ± 0.5%.

**Figure 5:**
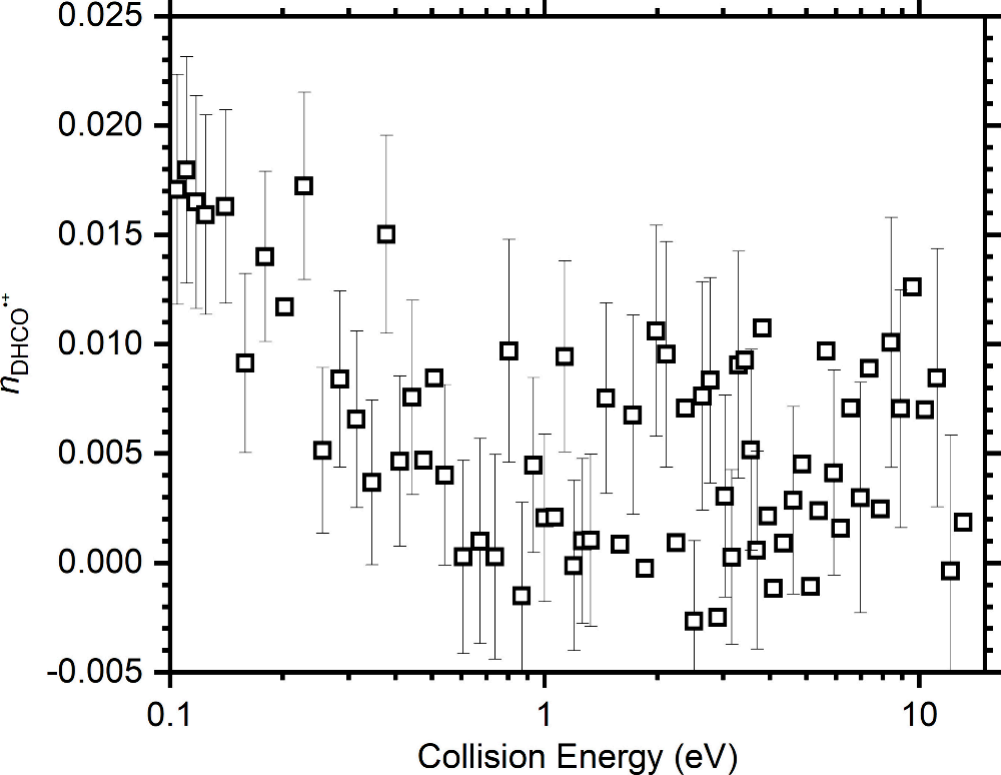
Isomeric fraction of DHCO^•+^, *n*_DHCO^•+^_, as a function of the collision energy. This is obtained from σ_*T*_, recorded at a photon energy of 13.07 eV, and σ_DHCO^•+^_, recorded at 11.00 eV, as detailed in the text.

**Table 1:** Measured σ_DHCO^•+^_ and σ_*T*_ Recorded at Different Collision Energies, along with *n*_DHCO^•+^_ Values Determined Using equation 4

Collision Energy (eV)	σ_DHCO^•+^_ (Å^2^)	σ_*T*_ (Å^2^)	*n*_DHCO^•+^_
0.16 ± 0.08	70.90 ± 0.69	0.65 ± 0.28	0.0092 ± 0.0041
2.0 ± 0.08	25.62 ± 0.34	0.27 ± 0.12	0.0105 ± 0.0048
11.0 ± 0.08	14.44 ± 0.25	0.12 ± 0.09	0.0083 ± 0.0064

Importantly, the mass spectra of the recorded product ions for the reaction of both H_2_CO^•+^ and the *m*/*z* 31 fragment of D_3_COH (see Figure S2 in the Supporting Information) indicate that the reactivity of the *m*/*z* 31 fragment is markedly different from that of the H_2_CO^•+^ ion. While detailed consideration of the various reaction processes is beyond the scope of this work, we focus here on the most intense channels from both parent ions. From H_2_CO^•+^ these are observed at *m*/*z* 58 (C_4_H_10_^•+^ from charge transfer) and at *m*/*z* 42 (C_3_H_6_^•+^ from dissociative charge transfer leading to the loss of CH_4_, exothermic by 0.30 eV^26,28^). By contrast, from the *m*/*z* 31 fragment from CD_3_OH (i.e., DCOH^•+^) the most intense products are at *m*/*z* 43 (C_3_H_7_^+^ from dissociative proton transfer leading to the loss of CH_4_, exothermic by 0.63 eV^26,28^), 57 (C_4_H_9_^+^ from dissociative proton transfer leading to the loss of H_2_, exothermic by about 0.84 eV^26,28^), and 32 (DHCOH^+^ formed via hydrogen atom transfer, exothermic by 0.77 eV^26,28^). As the reaction products for this ion are distinct from those of H_2_CO^•+^, we conclude that they arise from the reaction of a structurally distinct ionic species which, due to the lack of reasonable alternatives, must be DCOH^•+^.

As no photon energy dependence is observed for the recorded absolute reaction cross sections, we infer that the formation of DHCO^•+^ is energetically accessible from the threshold of the *m*/*z* 31 fragment channel and that the isomeric purity is therefore constant over the photon energy range considered here. We tentatively rationalize this observation as the result of efficient H/D scrambling via the interconversion between CD_3_OH^•+^ and the isotopic isomers D_2_COHD^•+^, D_2_HCOD^•+^ and DHCOD_2_^•+^, with the fragmentation step being the [1,1] ejection of D_2_ from DHCOD_2_^•+^. Further in-depth computational investigations are required, complementing the experimental measurements reported here, to allow for a detailed discussion of the different competing pathways. However, we note here that, due to the pathways via H/D exchange, we would expect the isomeric purity to be markedly lower in the case of nondeuterated methanol (CH_3_OH) due to the overlap between H_2_CO^•+^ and HCOH^•+^ fragmentation channels, thereby significantly removing the need for multiple rearrangements prior to fragmentation to give an isobaric contaminant.

In conclusion, we present experimental characterization of the HCOH^•+^ and DCOH^•+^ ions generated via the dissociative ionization of cyclopropanol and trideuterated methanol, respectively. In contrast to earlier suggestions,^37 ,38^ we find that cyclopropanol is unsuitable for this purpose. However, the generation of DCOH^•+^ from CD_3_OH is suitable, with an isomeric purity of 99.3 ± 0.5%. The key finding of this study is that DCOH^•+^ ions generated via the dissociative photoionization of trideuterated methanol (CD_3_OH) could be used in future studies to provide important isomer-specific reactivity data for various reaction systems. Such studies are not only essential for the development of accurate models of various astrochemical environments but should also allow for an understanding of the relationship between structure and reactivity that is highly valuable for fundamental reaction dynamics.

## Data Availability

Supporting data can be obtained from DataCat, the University of Liverpool Research Data Catalogue, at 10.17638/datacat.liverpool.ac.uk/2830.

## Supporting Information Available

Further details on the experimental methods used in this work as well as the data treatment methods used to correct the data presented in this work; data plots are also provided for which summaries are given in the text (PDF)
