# Supplementary material for: Experimental Characterization of the Isomer-Selective Generation of the Astrochemically Relevant Hydroxymethylene Radical Cation (HCOH•+/DCOH•+)
Source: J Phys Chem Lett. 2024 Oct 24;15(43):10888–95. doi: 10.1021/acs.jpclett.4c02374 (PMC11533223; doi:10.1021/acs.jpclett.4c02374)
Supplement: Supplementary file 1 — jz4c02374_si_001.pdf [file jz4c02374_si_001.pdf]

Experimental Characterization of the Isomer-Selective Generation of the Astrochemically Relevant Hydroxymethylene Radical Cation ($HCOH^{\bullet+}$/$DCOH^{\bullet+}$) - Supporting Information

Vincent Richardson,[*,†] Luke Alcock,[†] Nicolas Solem,[‡] David Sundelin,[¶] Claire Romanzin,[‡] Roland Thissen,[‡] Wolf D. Geppert,[¶] Christian Alcaraz,[‡] Miroslav Polášek,[§] Brianna R. Heazlewood,[†] Quentin Autret,[‡] and Daniela Ascenzi[‖]

†*Department of Physics, The Oliver Lodge, University of Liverpool, Oxford Street, Liverpool, UK L69 7ZE*

‡*Université Paris-Saclay, CNRS, Institut de Chimie Physique, UMR8000, 91405 Orsay, France/Synchrotron SOLEIL, L'Orme de Merisiers, 91190 Saint Aubin, France*

¶*Department of Physics, Stockholm University, Roslagstullsbacken 21, S-10691 Stockholm, Sweden*

§*J. Heyrovský Institute of Physical Chemistry of the Czech Academy of Sciences, Dolejššková 2155/3, 182 23 Prague, Czechia*

‖*Department of Physics, University of Trento, Via Sommarive 14, 38123 Trento, Italy*

E-mail: vincent.richardson@liverpool.ac.uk

S1. Experimental Methodology

All data on the reactivity of various species presented in this work has been collected using the CERISES apparatus[1,2] in conjunction with the DESIRS beamline[3] of the SOLEIL synchrotron in Saint-Aubin (France). CERISES is a guided ion beam tandem mass spectrometer (GIB-MS) consisting of two octopoles located between two quadrupole mass filters. In this way, reagent ions are mass selected prior to introduction into the scattering cell and product ions are mass selected prior to detection.

Neutral reagent pressures used were of the order of 3.0x10^{-8} bar in the case of C_2D_4 and 3.5x10^{-8} bar in the case of $CH(CH_3)_3$, in order to ensure operation close to the single collision regime. This reduces the contribution from secondary reactions while limiting parent beam attenuation to under 10%. Absolute pressures were measured using a MKS 398H differential manometer.

As detailed in the main text, all ions are generated by either direct or dissociative photoionization, with the photon energies used being in the range of 8.9-15 eV, with a resolution of 20-40 meV defined by the monochromator slit setting. Photons of energies greater than 15.7 eV are removed by an Argon gas filter operated at its nominal pressure of 2.3x10^{-4} bar.[4] Photon energies in the absolute scale were obtained using the absorption lines of Argon around 11.823 and 14.304 eV,[5,6] with systematic shifts of 15-27 meV above the tabulated values.

The collision energy available to the reactants depends on both the ionic charge (+1 in this case) and the potential difference between the ion source and scattering cell. The retarding potential method[7] has been used to determine the maximum of the first derivative of the parent ion yield, the corresponding voltage of which defines the zero of the kinetic energy in the laboratory frame, which can then be converted into centre-of-mass collision energies (E_{CM}). Collision energy ranges and FWHM of the distribution in kinetic energies of the reactant ion for the different reaction systems are given in Table S1.

In all cases, data were collected in the "multi-scan" mode where the signals for all ionic

Table S1: Collision energy ranges and FWHM for the different reaction systems studied as part of this work.

Reaction System	Minimum E_{CM} (eV)	Maximum E_{CM} (eV)	E_{CM} FWHM (eV)
$[CH_2O]^{\bullet+}/C_2H_6{}^{\bullet+}$ plus C_2D_4	0.07	10.39	0.05
$H_2CO^{\bullet+}$ plus $CH(CH_3)_3$	0.10	13.20	0.08
$[CDHO]^{\bullet+}$ plus $CH(CH_3)_3$	0.09	13.05	0.07

species of interest are collected at a given energy (either photon or collision) before moving to the next one. This is done to reduce the impact of any drifts in either source or reaction cell pressures.

S2. Data Treatment of Cross Sections for the Reaction of *m/z* 30 Fragments of Cyclopropanol with C_2D_4

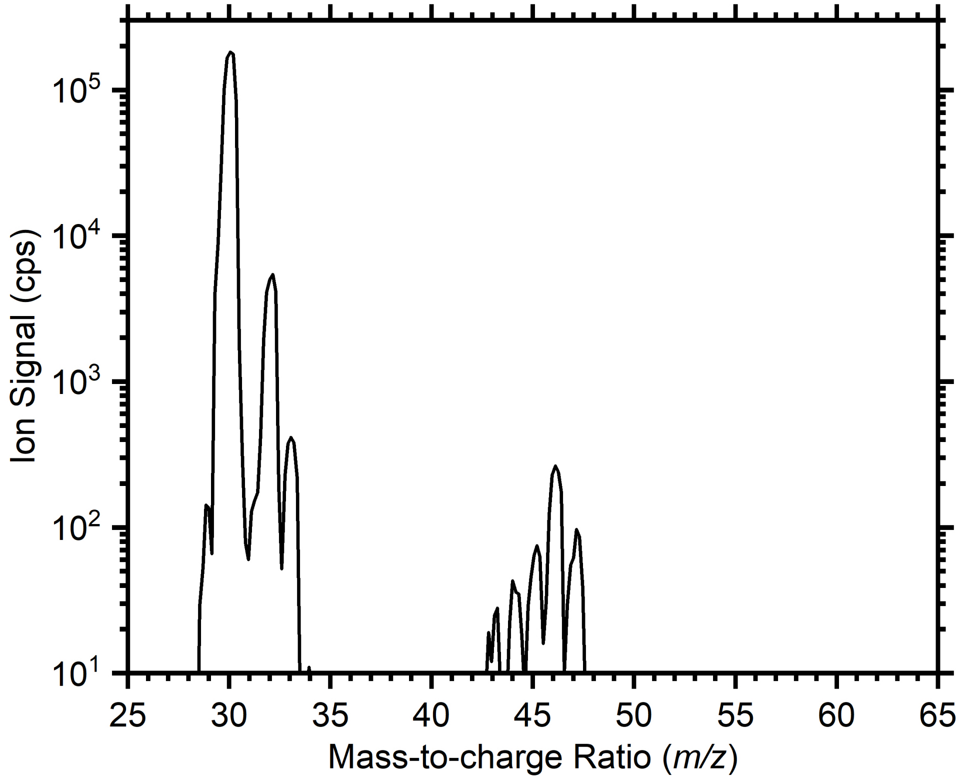

Figure S1: Mass spectra of the products of the reaction of *m/z* 30 fragment ion of cyclopropanol, formed at a photon energy of 11.2 eV, with C_2D_4 at a collision energy of 0.17 eV.

In order to obtain accurate absolute reaction cross sections for the charge transfer reactions, a series of corrections have been made. Firstly, in instances where products have been identified at adjacent masses, the cross section of the higher mass product has been corrected to account for the ^{13}C contribution from the lower mass channel. A representative mass spectrum for this reaction prior to ^{13}C correction is shown in Figure S1. As none of the charge transfer products have an adjacent lower mass product channel of significance, this correction has no impact on the measured charge transfer cross section.

The second is to account for a number of secondary reactions of the *m/z* 32 product ions, which arise from the fact that the primary products from charge transfer (as well as proton

transfer when this channel is present) have low velocities in the laboratory frame and are therefore likely to undergo secondary reactions with neutrals in the scattering cell. This is well known issue in guided ion beam experiments. From literature, we are able to identify the most probable and significant secondary reaction products as being those observed at m/z 46 and 62,[8–10] with tertiary products at m/z 74 and 78.[10,11] The equations for these reactions are as follows:

$$C_2D_4^{\bullet +} + C_2D_4 \rightarrow C_3D_5^+ + CD_3 \qquad (m/z\,46) \qquad (S1)$$

$$\rightarrow C_4D_7^+ + H \qquad (m/z\,62) \qquad (S2)$$

$$C_3D_5^+ + C_2D_4 \rightarrow C_5D_7^+ + H_2 \qquad (m/z\,74) \qquad (S3)$$

$$\rightarrow C_5D_9^+ \qquad (m/z\,78) \qquad (S4)$$

The extent of this correction varies with collision energy, but is broadly on the order of a 5-15% increase with respect to the m/z 32 mass channel, with this almost entirely arising from the m/z 46 product channel.

Finally, in order to correct for the decrease in observed ion signal, and therefore absolute cross section, at low collision energies due to the loss of backward scattered products in the laboratory frame, cross sections have been manually corrected to give the absolute cross section values as a function of the collision energy given in Figure 2 of the main paper. This is done by increasing the cross sections at low collision energies by 25% to give a linear trend over the full collision energy range.

Reactions with Isobutane ($CH(CH_3)_3$)

Representative mass spectra for the reaction of both $H_2CO^{\bullet+}$ from formaldehyde and the m/z 31 fragment from CD_3OH with isobutane are shown in Figure S2. We note the significant difference in the range of products, and corresponding relative intensities, for the two different ions. Full analysis of the overall chemistry of this systems is beyond the scope of this work, and calibration of the isomeric purity is performed exclusively through consideration of the charge transfer product at m/z 58.

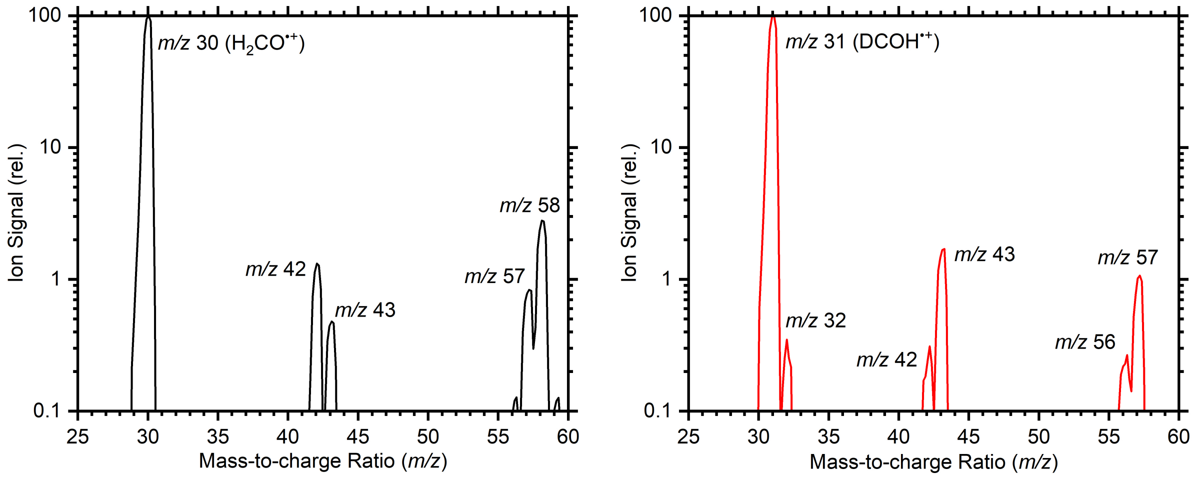

Figure S2: Mass spectra of the products of the reaction of both $H_2CO^{\bullet+}$ (*left*) and $DCOH^{\bullet+}$ (*right*) with isobutane, $CH(CH_3)_3$. Reactions have been performed at photon energies of 11.00 and 13.07 eV, respectively, and collision energies of 0.18 and 0.17 eV, respectively.

Absolute reaction cross sections for the m/z 58 charge transfer product ($CH(CH_3)_3{}^{\bullet+}$) of the reaction of both $H_2CO^{\bullet+}$ and the m/z 31 fragment of CD_3OH with isobutane are shown in Figure 5 in the main text. The step function visible in CS for both channels at a collision energy of ∼0.9 eV is the due to the "L3 effect", a key indicator of a charge transfer process. This is because the reaction kinematics of charge exchange processes, occurring with very limited momentum transfer to the neutral reactant, is such that, at low collision energies, while forward scattered products with nonzero velocity will drift to the end of the octupole where they will be extracted and detected, backward scattered products in the lab frame will be lost due to the attractive voltage exerted by the injection lens (L3) into the octupole.[12]

This leads to an under-observation of such product channels at low collision energies.

S3. Further Experimental Data

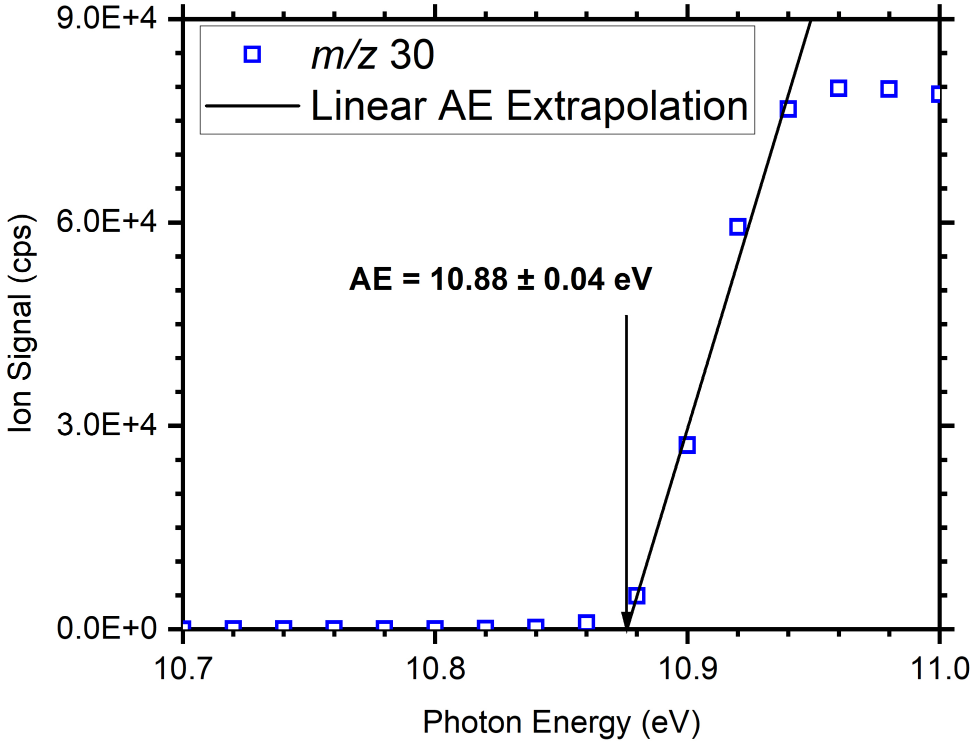

Figure S3: PIE curves for the photoionization of H_2CO giving H_2CO^+ (m/z 30). The AE shown has been determined via linear threshold extrapolation of the photoionization efficiency (PIE) curve.[13–15]

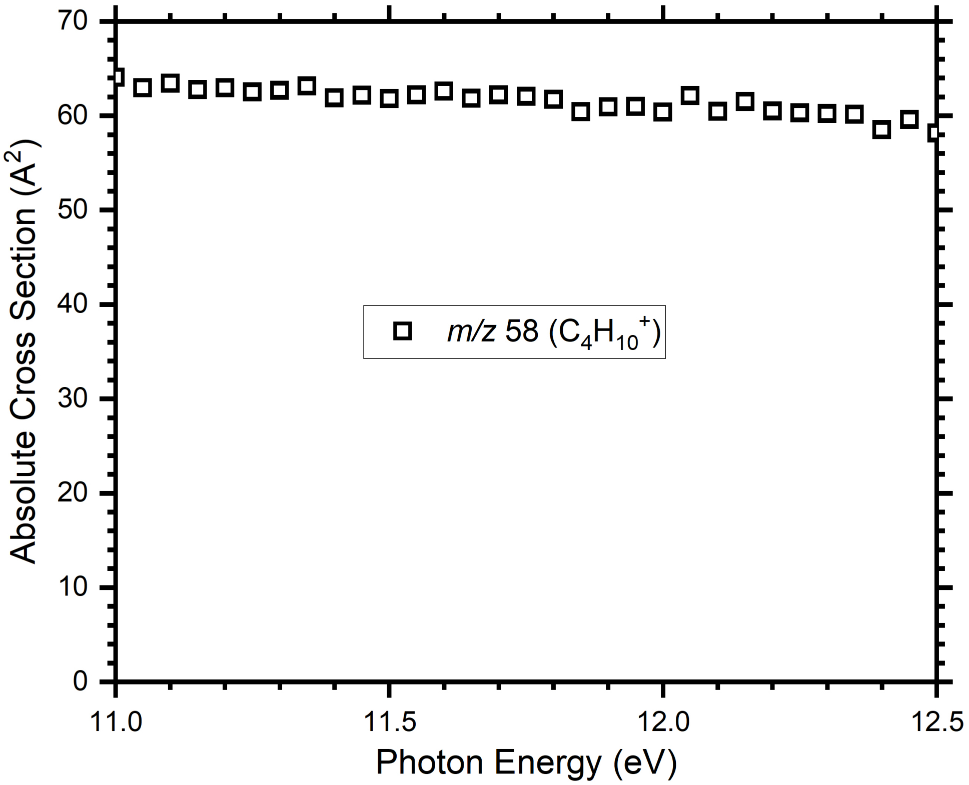

Figure S4: Absolute cross sections as a function of the photon energy for the m/z 58 product of the reaction of $H_2CO^{\bullet +}$ with $CH(CH_3)_3$ at a collision energy of 0.18 eV. Error bars shown correspond to the statistical error determined through the data treatment.

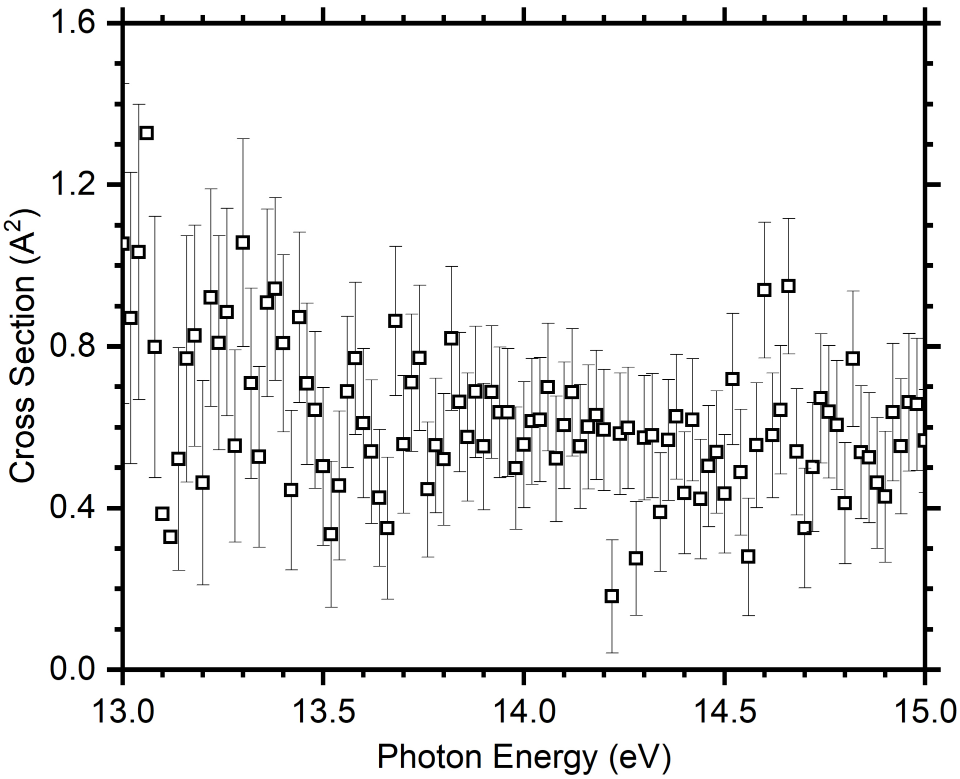

Figure S5: Absolute cross sections as a function of the photon energy for the *m/z* 58 product of the reaction of $[CDHO]^{\bullet +}$ from CD_3OH with $CH(CH_3)_3$ at a collision energy of 0.17 eV.

References

(1) Alcaraz, C.; Nicolas, C.; Thissen, R.; Žabka, J.; Dutuit, O. $^{15}N^+$ + CD_4 and O^+ + $^{13}CO_2$ State-Selected Ion-Molecule Reactions Relevant to the Chemistry of Planetary Ionospheres. *Journal of Physical Chemistry A* **2004**, *108*, 9998–10009.

(2) Cunha de Miranda, B.; Romanzin, C.; Chefdeville, S.; Vuitton, V.; Žabka, J.; Polásek, M.; Alcaraz, C. Reactions of State-Selected Atomic Oxygen Ions O^+(4S, 2D, 2P) with Methane. *Journal of Physical Chemistry A* **2015**, *119*, 6082–6098.

(3) Nahon, L.; de Oliveira, N.; Garcia, G.; Gil, J.-F.; Pilette, B.; Marcouilleé, O.; Lagarde, B.; Polack, F. [DESIRS]: a state-of-the-art VUV beamline featuring high reso-

lution and variable polarization for spectroscopy and dichroism at SOLEIL. *Journal of Synchrotron Radiation* **2012**, *19*, 508–520.

(4) Mercier, B.; Complin, M.; Prevost, C.; Bellec, G.; Thissen, R.; Dutuit, O.; Nahon, L. Experimental and theoretical study of a differentially pumped absorption gas cell used as a low energy-pass filter in the vacuum ultraviolet photon energy range. *Journal of Vacuum Science and Technology* **2000**, *18*, 2533–2541.

(5) Minnhagen, L. Spectrum and the energy levels of neutral argon, Ar. *Journal of the Optical Society of America* **1973**, *63*, 1185–1198.

(6) Linstrom, P. J.; Mallard, W. G. NIST Chemistry WebBook - Standard Reference Database n. 69. [Online], accessed May 2024; `http://webbook.nist.gov`.

(7) Teloy, E.; Gerlich, D. Integral cross sections for ion-molecule rections. I. The guided beam technique. *Chemical Physics* **1974**, *4*, 417–427.

(8) Anicich, V.; McEwan, M. Ion-molecule chemistry in Titan's ionosphere. *Plan. Space Sci.* **1997**, *45*, 897–921.

(9) McEwan, M.; Scott, G.; Anicich, V. Ion-molecule reactions relevant to Titan's ionosphere. *Int. J. Mass Spectrom. Ion Proc.* **1998**, *172*, 209–219.

(10) Anicich, V. G. An index of the literature for bimolecular gas phase cation-molecule reaction kinetics. 2003; `https://hdl.handle.net/2014/7981`.

(11) Anicich, V.; Wilson, P.; McEwan, M. Termolecular ion-molecule reactions in Titan's atmosphere. IV. A search made at up to micron in pure hydrocarbons. *J. Am. Chem. Soc. Mass Spectrom.* **2003**, *14*, 900–915.

(12) Ervin, K.; Armentrout, P. Translational energy dependence of $Ar^+ + XY \rightarrow ArX^+ + Y$ (XY = H_2, D_2, HD from thermal to 30 eV c.m. *J. Chem. Phys.* **1985**, *83*, 166–189.

(13) Chupka, W. Effect of Thermal Energy on Ionization Efficiency Curves of Fragment Ions. *J. Chem. Phys.* **1971**, *54*, 1936–1947.

(14) Traeger, J.; McLoughlin, R. Absolute heats of formation for gas-phase ions. *J. Am. Chem. Soc.* **1981**, *103*, 3647–3652.

(15) Castrovilli, M.; Bolognesi, P.; Cartoni, A.; Catone, D.; O'Keefe, P.; Casavola, A.; Turchini, S.; Zema, N.; Avaldi, L. Photofragmentation of Halogenated Pyrimidine Molecules in the VUV Range. *J. Am. Soc. Mass Spectrom.* **2014**, *25*, 351–367.
